# Burnout among Residents: Prevalence and Predictors of Depersonalization, Emotional Exhaustion and Professional Unfulfillment among Resident Doctors in Canada

**DOI:** 10.3390/ijerph20043677

**Published:** 2023-02-19

**Authors:** Reham Shalaby, Folajinmi Oluwasina, Ejemai Eboreime, Hany El Gindi, Belinda Agyapong, Marianne Hrabok, Sumeet Dhanoa, Esther Kim, Izu Nwachukwu, Adam Abba-Aji, Daniel Li, Vincent Israel Opoku Agyapong

**Affiliations:** 1 Department of Psychiatry, University of Alberta, Edmonton, AB T6G 2R3, Canada; 2 Department of Critical Care Medicine, King Abdul-Aziz Hospital, Jeddah 22421, Saudi Arabia; 3 Department of Psychiatry, University of British Columbia, Vancouver Island, BC V6T 1Z4, Canada; 4 Department of Psychiatry, University of Calgary, Calgary, AB T2N 1N4, Canada; 5 Alberta Health Services, Edmonton, AB T5J 3E4, Canada; 6 Department of Psychiatry, Dalhousie University, Halifax, NS B3H 2E2, Canada

**Keywords:** burnout, residents, cross-sectional, online survey, work hours

## Abstract

Background: Burnout in the medical profession has garnered a lot of attention over recent years. It has been reported across all specialties and all stages of medical education; however, resident doctors in particular are at risk for burnout throughout their years of training. This study was aimed at evaluating the prevalence and correlates of burnout among resident doctors in Alberta. Methods: Through a descriptive cross-sectional study design, a self-administered questionnaire was used to gather data from resident doctors at two medical schools in Alberta, Canada. The Maslach Burnout Inventory was used as the assessment tool. Chi-squared and multivariate binary logistic regression analyses were used. Results: Overall burnout prevalence among residents was 58.2%, and for professional fulfilment index, it was 56.7% for work exhaustion and interpersonal disengagement and 83.5% for lack of professional fulfillment. Working more than 80 h/week (OR = 16.437; 95% CI: 2.059–131.225), being dissatisfied (OR = 22.28; 95% CI: 1.75–283.278) or being neither satisfied nor dissatisfied with a career in medicine (OR = 23.81; 95% CI: 4.89–115.86) were significantly associated with high depersonalization. Dissatisfaction with efficiency and resources (OR = 10.83; CI: 1.66–70.32) or being neither satisfied nor dissatisfied with a career in medicine (OR = 5.14; CI: 1.33–19.94) were significantly associated with high emotional exhaustion. Working more than 80 h/week (OR = 5.36; CI: 1.08–26.42) and somewhat agreeing that the residency program has enough strategies aimed at resident well-being in place (OR = 3.70; CI: 1.10–12.46) were significantly associated factors with high work exhaustion and interpersonal disengagement. A young age of residents (≤30 years) (OR = 0.044; CI: 0.004–0.445) was significantly associated with low professional fulfillment. Conclusion: Burnout is a serious occupational phenomenon that can degenerate into other conditions or disrupt one’s professional performance. Significant correlates were associated with high rates of burnout. Leaders of medical schools and policymakers need to acknowledge, design, and implement various strategies capable of providing continuous effective mental health support to improve the psychological health of medical residents across Canada.

## 1. Introduction

Burnout in the medical profession is a topic that has garnered much attention over recent years. Burnout and associated mental health correlates, such as depression, suicidal ideation, and work dissatisfaction, have been reported across all specialties, and all stages of medical education and clinical practice [1]. The high levels of burnout are usually associated with decreased job satisfaction and performance, and poor quality of life [2,3,4]. It is believed that the seeds of burnout are planted as early as medical school, peak during residency training, and remain high when physicians face challenges related to their professional practice [1,5].

The term burnout, as currently conceptualized, was initially introduced in the early 1970s by psychoanalyst Freudenberger, and was later defined by Christina Maslach as a syndrome characterized by the triad of high emotional exhaustion, high depersonalization, and a low sense of personal accomplishment [6,7]. Burnout is the outcome of escalated unchecked professional and emotional distress in the context of workplace dissatisfaction [8]. It develops in individuals whose occupation entails contact with people, with emotional exhaustion appearing first and psychological isolation later, resulting in cynicism and detachment in interpersonal relations as a coping strategy [2]. The experience of burnout encompasses both physical and psychological dimensions, including but not limited to insomnia, appetite changes, irritability, and headaches. Although its symptom profile can mimic clinical depression, burnout is primarily related to the work environment rather than a global affective disturbance as seen in depression [2].

In general, burnout rates among medical professionals are disproportionately high compared to other populations. According to Shanafelt et al., the incidence of burnout symptoms among US physicians was 37.9%, compared to 27.8% in a control sample population of non-physicians [9]. Similarly, healthcare professionals working in the field of mental health were at relatively high levels of stress and burnout [10]. In a narrative review examining burnout among residents and medical students, the authors reported that during residency the prevalence of high emotional exhaustion approximated 50%, depersonalization approximated 33%, and overall burnout was 60% [11]. Burnout rates are reportedly twice as high for residents in training compared to their non-medical postgraduate counterparts [12].

According to the Medscape National Physician Burnout and Suicide Report, the burnout rate among physicians was reported to be as high as 42% in 2020, compared with 46% and 39% in 2015 and 2013, respectively [6,13]. Similar high rates were reported for residents with scores between 41% and 74% across multiple specialties [1]. According to the Medscape Residents Lifestyle and Happiness Report, 2020, 27% of residents stated that they rarely or never had time to lead a satisfying social life; of these, 68% reported having failed relationships for this reason [14]. Furthermore, these figures increased in 2021 during the COVID-19 pandemic [15]; 35% of a sample of residents at the Emergency Department demonstrated symptoms of post-traumatic stress disorder, acutely during the COVID-19 pandemic crisis [16]. Similarly, about 60% of medical professionals, including medical residents in Canada, have stated that their mental health worsened after the pandemic [17].

An increased susceptibility of residents to experience burnout during their years of training has been attributed to several factors. It has been documented that this period naturally entails substantial duties including excessive working hours, call requirement obligations, sleep deprivation, loss of autonomy, and lack of control over one’s schedule [2,3,4]. The emotional demands and poor environmental factors usually render residents routinely challenged with high demands, imbalanced work and home life, low autonomy and burnout [2,3,4]. Inadequate wages and high amounts of educational debt have also been found to result in burnout among residents [2,18]. In a large study that included about 75% of all internal medicine residents in the USA, the authors concluded that increasing educational debt was associated with lower quality of life and high burnout rates, and was ultimately related to poor training outcomes and low scoring on examinations [18].

The experience of burnout has the potential to jeopardize not only the well-being of residents but also the care and safety of their patients. The risk of depression, stress, alcohol use, unprofessional behaviors, negative relationships, and suicidality were increasingly reported among residents who have experienced burnout, less sleep time and more working hours [1,19,20]. Additionally, it was reported that physicians with high levels of distress and burnout are more prone to self-perceive or reportedly commit medical errors [1,19,21]. This may occur through the negative impact of lack of sleep, distress, absenteeism, and long working hours on commitment, professionalism, medical knowledge, competency, and attention to detail [1,18,20]. On the other hand, studies have recognized that physicians who have high empathy tend to demonstrate positive attributes, with better clinical outcome profiles in terms of achieving better relationships with high patient satisfaction and moral reasoning with clinical competence [22,23,24].

A number of factors have been examined in the literature as potential correlates of burnout among residents. These included sociodemographic factors such as gender and age, country of residency, and country of original medical training, or residency-related factors such as training specialty, the year of residency, or perceived low provided patient care [11,18,25]. However, the results were essentially non-conclusive; for example, while some studies suggest the prevalence of burnout may either increase [26] or decline with the progression in residency [27], other studies suggested that it has a similar trend across the years of training [28], or otherwise it may maximize during a certain year, such as year two of residency [29].

A dearth of research has been run, however, exploring the levels of burnout or comparing the rates among medical residents in Alberta, a western province of Canada with a population of 4,286,134 in 2017 [30]. Along with the non-conclusive information related to the burnout correlates, there was an essential need to establish recent information with updated knowledge in this field and to identify any potential correlates with burnout phenomenon, hoping to provide effective interventions that can be implemented to promote health and wellness among medical residents in Alberta.

In this study, we aimed to elaborate further on the burnout phenomenon among medical residents in Alberta, and to explore the potential related factors. The objective was to evaluate the prevalence of burnout among resident doctors in Alberta and identify their correlates with individual and residency-related factors.

## 2. Methods

### 2.1. Study Setting and Design

This study followed the published protocol [31] aiming to address the prevalence and correlates of burnout and professional fulfillment among resident physicians in Alberta, a western province of Canada with a population of 4,286,134 in 2017 [30]. As of December 2018, there were 10,674 physicians registered in the province by the College of Physicians and Surgeons of Alberta, Canada; of them, there were 2742 educational registrants [32].

Through a descriptive cross-sectional study design, a self-administered, anonymous online questionnaire was used. Respondents were resident doctors at the time of this study who had been attending the University of Alberta, Faculty of Medicine and Dentistry, and the University of Calgary, Cumming School of Medicine participated in this study. Data collection took place in the period between 1st of October 2020 and 31st October 2020.

### 2.2. Study Hypothesis

We hypothesized that the prevalence of burnout among Albertan residents will be high and closely associated with certain socio-demographic characteristics and work-related factors.

### 2.3. Institutional Review Board Approval

The study was conducted following the Declaration of Helsinki (Hong Kong Amendment) and Good Clinical Practice (Canadian Guidelines). All participants were provided with an online information leaflet, and informed consent was obtained before participation. The study received ethical approval from the Health Ethics Research Board of the University of Alberta (reference number Pro00091436) and the Conjoint Health Research Ethics Board of the University of Calgary (REB19-1457).

### 2.4. Data Collection and Outcome Measure

Data collection tools for this study were developed based on the published literature and questions from previously validated instruments. The general constructs of interest included relevant demographic information, current practice and career planning, general health status, mental health status, and rates of burnout, as well as factors contributing to both burnout and resilience among respondents. In addition, the survey included an open-ended question to facilitate qualitative data collection. Standardized measures from which questions were selected and included in the survey were the Maslach Burnout Inventory (MBI), the Canadian Medical Association National Physician Health Survey, the Mini Z burnout survey, and the Professional Fulfillment Index [33,34,35,36,37,38].

The MBI, a short questionnaire-based tool, was designed to measure the symptoms and severity of burnout, representing the main outcome of this study. Three main domains were examined: depersonalization, emotional exhaustion, and professional fulfilment index (PFI). PFI includes two domains: work exhaustion and interpersonal disengagement (Burnout scale) and professional fulfillment scale. These standardized measures provided information for the study results.

For burnout scores, a Likert scale from 0 (never) to 6 (every day) was used. “High emotional exhaustion:” is screened positive if >3 “positive screen” and negative if less than 3 “negative screen”. Similarly, high depersonalization or (i.e., lower interpersonal empathy) is screened positive if >3: “positive screen”, while it was considered negative if less than = 3 “negative screen”.

For PFI, items are scored 0 to 4. Each dimension is treated as a continuous variable. Scale scores are calculated by averaging the item scores of all the items within the corresponding scale. Scale scores can then be multiplied by 25 to create a scale range from 0 to 100. A higher score on the professional fulfilment scale is more favorable. In contrast, higher scores on the work exhaustion and interpersonal disengagement scales are less favorable.

Dichotomous PFI burnout categories are determined from the average item score (range 0 to 4) of all 10 burnout items (work exhaustion and interpersonal disengagement), using a cut-point of 1.33. Dichotomous professional fulfilment is recommended at an average item score cut-point of >3.0. For overall burnout prevalence, respondents were considered to have burnout if they have high scores on emotional exhaustion or depersonalization subscales.

The reliability of MBI is supported by several studies, where Cronbach alpha ratings are 0.90 for emotional exhaustion, 0.76 for depersonalization, and 0.76 for personal accomplishment [39].

Sections (subscales) of different scales for measuring burnout were selected and used in this study in order to measure different dimensions of burnout which were of interest to the researchers. We also adopted the MBI subscales for use in determining the overall burnout state of respondents, as the MBI is the most widely used scale for the measurement of burnout and would allow for comparison of this study with the published literature.

### 2.5. Sample Size

At the time of this study, a total number of 1594 resident doctors were registered across all specialties at the Universities of Alberta and Calgary [40]. An anticipated sample size of 959 was determined based upon a 95% confidence level and a margin of error of 2% for prevalence rate estimates for resident doctors’ burnout.

### 2.6. Statistical Analysis

Data analysis was undertaken using SPSS Statistics for Windows (Version 26; IBM Corp, Armonk, NY, USA) [41]. Demographic characteristics of residents, as well as responses to questions related to professional satisfaction, workplace collegiality, and support, were summarized by absolute numbers and percentages. Two age categories were created out of the age continuous variable using 30 years as a cut-off score. We hypothesize that residents who are less than 30 years old may express fewer overall burnout symptoms compared to the older group. Similarly, we used the cut-off score of 80 h work/week, since from previous research working more than this amount of time could be a risk factor for burnout symptoms [42,43,44].

Only completed responses were reported, with no data imputation. Chi-square/Fisher’s exact analysis with two-tailed significance (*p* ≤ 0.05) was performed to assess the association between the demographic, professional satisfaction, workplace collegiality, and support variables of residents and responses to questions related to burnout and professional fulfillment. Variables with statistically significant or near significant association (*p* ≤ 0.1) for each of the domains of burnout and professional fulfillment were entered into their respective multivariate binary logistic regression models, predicting the likelihood of the four outcome variables (depersonalization, emotional exhaustion, work exhaustion and interpersonal disengagement, and professional fulfillment). Before performing logistic regression analysis, correlational diagnostics were performed to identify any strong inter-correlations (Spearman’s correlation coefficient of 0.7 to 1.0 or −0.7 to −1.0) among predictor variables. Odds ratios from the binary logistic regression analysis were calculated to determine the association between the predictor variables and the presence of burnout or professional fulfillment domain, controlling for the other variables in each model.

## 3. Results

Out of the 1594 resident doctors reached in Alberta with the survey link, there were 157 responses received (response rate: 9.8%), of which 140 responses were complete. Only partially and fully completed responses were included in the analysis.

Table 1, below, illustrates the distribution of the socio-demographic information as well as the academic factors of the respondents.

Of the residents who participated in the survey, the majority were aged less than or equal to 30 years (59.3%), female (60.7%), did not have dependents (72%), and were in a relationship (77.9%).

This study showed a predominantly Caucasian ethnicity of the respondents (65.7%). Most residents owed less than USD 100,000 (52.1%), while the rest owed more than USD 100,000 in education loans. The majority were in their first and second postgraduate year (25.7% and 24.3%), respectively. Just above a quarter of the respondents worked more than 80 h per week (25.7%). The majority were part of the family medicine and internal medicine residency programs (23.6% each).

Table 1 also shows the descriptive statistics of professional satisfaction, workplace collegiality, and support. Most respondents were satisfied with the quality of peer collaboration with their colleagues (75.9%), satisfied with their quality of interaction with attending physicians (76.7%), satisfied with the quality of learning environment (69.2%), and satisfied with their workload and job demand (45.1%).

Most residents were dissatisfied with control and flexibility (53.4%) and with work–life integration (42.9%), while 38.3% of the residents were neither satisfied nor dissatisfied with the efficiency and resources available to them.

There was predominantly agreement among residents regarding workplace collegiality variables. The majority agreed that they were satisfied with their career in medicine (62%), find their colleagues to be very supportive (83%), find people treat each other with respect (78.3%), find a spirit of cooperation and teamwork exists in their group (78.3%), and find disputes or conflicts are resolved fairly in their work groups (63.6%).

Regarding support variables, most residents felt well supported when they reached out to their friends and family (87.5%). Residents described what would occur if they reached out for help to those in their learning and work environment as somewhat or very supportive (78.3%), and somewhat agreed that their residency program had enough strategies aimed at residents’ well-being in place (38.3%).

With respect to the burnout rates among respondents, four variables examined the presence of burnout, depersonalization (40.3%), emotional exhaustion (48.1%), work exhaustion and interpersonal disengagement (56.7%), and absent professional fulfilment (83.5%).

The overall burnout prevalence was 58.2%, since respondents who had high scores on emotional exhaustion or depersonalization subscales were (75/129).

### 3.1. Univariate Analysis of Burnout Variables

(1) Depersonalization

The univariate analysis in Table 2 included 24 demographic burnout variables in relation to the presence of depersonalization. A Chi-squared or Fisher exact test revealed a significant association between the presence of depersonalization and 14 variables, including current residency program you are part of, total working hours work per week (clinical and non-clinicals), quality of interaction with your attending physicians, quality of learning environment, workload and job demand, control and flexibility, work–life integration (meeting personal and professional obligations), efficiency and resources, overall satisfaction about pursuing a career in medicine, if they find their colleagues to be supportive, disputes or conflicts are resolved fairly in their work group, how well they feel supported by their social support/friends/family, how they would best describe what would occur if they reached out for help to those in their learning and work environment, and if they feel that their residency program had enough strategies aimed at residents’ well-being in place.

(2) Emotional Exhaustion

The Chi-square test in Table 2 showed a significant (p ≤ 0.05) relationship between emotional exhaustion and 14 sociodemographic, professional satisfaction and support variables, such as total hours of work per week (clinicals and non-clinicals), quality of peer collaboration among residents colleagues, quality of interaction with their attending physicians, quality of their learning environment, workload and job demand, control and flexibility, work–life integration (meeting personal and professional obligations), efficiency and resources, overall satisfaction about pursuing a career in medicine, if they find their colleagues to be supportive, if a spirit of cooperation and teamwork exists in their work group, disputes or conflicts are resolved fairly in their work group, how they would best describe what would occur if they reached out for help to those in their learning and work environment, and if they feel their residency program had enough strategies aimed at residents’ well-being in place.

(3) Professional Fulfillment Index:
  (a) Work Exhaustion and Interpersonal Disengagement:
  The Chi-square test in Table 3 showed a significant (*p* ≤ 0.05) relationship between work exhaustion and interpersonal disengagement and 11 administrative, professional satisfaction and support variables, including total hours they work per week (clinical and non-clinical), quality of interaction with their attending physicians, quality of their learning environment, workload and job demand, control and flexibility, work–life integration (meeting personal and professional obligations), efficiency and resources, overall satisfaction about pursuing a career in medicine, people treat each other with respect in their work group, disputes or conflicts are resolved fairly in their work group, and if they feel that their residency program had enough strategies aimed at residents’ well-being in place.
  (b) Professional Fulfillment Scale:
  The univariate analysis in Table 3 also showed the association between professional fulfillment and nine socio-demographic, workplace collegiality and support variables, such as age, quality of their learning environment, workload and job demand, control and flexibility, work–life integration (meeting personal and professional obligations), efficiency and resources, their overall satisfaction about pursuing a career in medicine, disputes or conflicts are resolved fairly in their work group, and if they feel that their residency program had enough strategies aimed at residents’ well-being in place.

### 3.2. Multivariable Binary Logistic Regression Analysis

Table 4 summarizes the significant results of the logistic regression analysis models predicting the likelihood of depersonalization, emotional exhaustion, work exhaustion and interpersonal disengagement, and professional fulfillment.

#### 3.2.1. Depersonalization

The multivariate logistic regression model included 13 out of 14 chi-squared predictor variables; one variable was removed, “work–life integration (meeting personal and professional obligations)”, that showed a high correlation (r_s_ > 0.7) with another variable: “workload and job demand”. A detailed model is illustrated in Appendix A: Table A1.

The model was statistically significant; *Χ*^2^ (df = 27; n = 120) = 67.82, *p* < 0.001, suggesting that the model could distinguish between respondents who likely have high depersonalization or low depersonalization among resident doctors. The model accounted for 43.2% (Cox and Snell R^2^) to 58.7% (Nagelkerke R^2^) of the variance. According to the goodness-of-fit statistic using the Hosmer–Lemeshow goodness-of-fit test, the model was adequately fit (Chi^2^= 5.444; *p* = 0.709) and correctly classified 82.5% of cases.

Two variables significantly predicted depersonalization, working hours and satisfaction with a career in medicine. Residents who worked above 80 h per week were 16 times more likely to show depersonalization (OR = 16.437; 95% CI: 2.059–131.225) than those who worked less than 80 h per week, while controlling for other variables in the model.

Residents who were dissatisfied with their career in medicine were 22 times more likely to experience depersonalization (OR = 22.28; 95% CI: 1.75– 283.278) than those who agreed that they were satisfied with their career in medicine. Similarly, residents who neither agreed nor disagreed that they were satisfied with their career in medicine were 23 times more likely to show depersonalization (OR = 23.81; 95% CI: 4.89–115.86) compared to those who agreed that they were satisfied with their career in medicine, while controlling for other variables in the model.

#### 3.2.2. Emotional Exhaustion

The regression model was employed to predict the likelihood of emotional exhaustion among resident doctors. The model included 13/14 chi-squared chi predictor variables, including one variable which was near significant. One variable was removed; “workload and job demand” showed a high correlation (r_s_ > 0.7) with another variable, “work–life integration”. The model was statistically significant; Χ^2^ (df = 25; n = 120) = 74.71, *p* < 0.001, suggesting that the model could distinguish between respondents who likely had high emotional exhaustion or low emotional exhaustion among resident doctors. The model accounted for 46.3% (Cox and Snell R^2^) to 61.9% (Nagelkerke R^2^) of the variance. A detailed model is illustrated in Appendix A: Table A2.

According to the goodness-of-fit statistic using the Hosmer–Lemeshow goodness-of-fit test, the model was adequately fit (Chi^2^= 3.07; *p* = 0.93) and correctly classified 81.7% of cases.

As shown in Table 4, only two variables independently predicted the likelihood of emotional exhaustion, overall satisfaction with a career in medicine, and efficiency and resources variables.

Resident doctors who were neither satisfied nor dissatisfied with their career in medicine were five times more likely to have emotional exhaustion (OR = 5.14; CI: 1.33- 19.94) than the ones who were satisfied with their career in medicine.

Resident doctors who were dissatisfied with the efficiency and resources were 11 times more likely to experience high emotional exhaustion than ones who were satisfied with the efficiency and resources (OR = 10.83; CI: 1.66- 70.32).

#### 3.2.3. Professional Fulfillment Index

(a) Work Exhaustion and Interpersonal Disengagement

The regression model included 11 out of 12 chi squared predictor variables, including one variable which was near significant. One variable was removed; “work–life integration (meeting personal and professional obligations)” showed high correlation (r_s_ > 0.7) with another variable, “workload and job demand”.

The model was statistically significant; *Χ*^2^ (df = 21; n = 120) = 54.25, *p* < 0.001, suggesting that the model could distinguish between respondents who were likely to experience work exhaustion and interpersonal disengagement from those who were not among resident doctors. The model accounted for 36.4% (Cox and Snell R^2^) to 48.7% (Nagelkerke R^2^) of the variance. A detailed model is illustrated in Appendix A: Table A3.

According to the goodness-of-fit statistic using the Hosmer–Lemeshow goodness-of-fit test, the model was adequately fit (Chi^2^= 4.520; *p* = 0.718) and correctly classified 79.2% of cases.

As shown in Table 4, two variables independently predict the likelihood of work exhaustion and interpersonal disengagement, the total number of hours worked per week, and the feeling of the residency program having enough strategies aimed at resident well-being in place.

Resident doctors who worked more than 80 h per week are five times more likely to experience work exhaustion and interpersonal disengagement (OR = 5.36; CI: 1.08- 26.42) than resident doctors who work less than 80 h per week.

Resident doctors who somewhat feel that their residency program had enough strategies aimed at resident well-being in place were about four times more likely to experience work exhaustion and interpersonal disengagement than ones who believed that their residency program had enough strategies aimed at residents’ well-being in place (OR = 3.70; CI: 1.10–12.46).

(b) Professional Fulfillment

The regression model included 10 out of 11 chi-squared predictor variables, including one variable which was near significant. One of the variables was removed; “work–life integration (meeting personal and professional obligations)” showed a high correlation (r_s_ > 0.7) with another variable, “workload and job demands.” The model was statistically significant; Χ^2^ (df = 21; n = 120) = 62.42, *p* < 0.001, suggesting that the model could distinguish between respondents who were low professionally fulfilled and high professionally fulfilled among the resident doctors. The model accounted for 40.6% (Cox and Snell R^2^) to 68.3% (Nagelkerke R^2^) of the variance.

According to the goodness-of-fit statistic using the Hosmer–Lemeshow goodness-of-fit test, the model was adequately fit (Chi^2^= 1.67; *p* = 0.989) and correctly classified 91.7% of cases.

Table 4 shows that there was only one variable which predicted the likelihood of the lack of professional fulfilment: the age of the participant residents. Resident doctors who were equal to or less than 30 years old were about 23 times more likely to experience low professional fulfilment than those who were above 30 years old (OR = 22.73; CI: 2.25–250). A detailed model is illustrated in Appendix A: Table A4.

## 4. Discussion

### 4.1. Principal Findings

This study presents the results of a cross-sectional survey designed to examine the prevalence and predictors of burnout among medical residents in Alberta, Canada. Principal findings showed that at least half of the residents endured burnout symptoms in at least one domain of the used scale (ranging between 40.3% for depersonalization and 83.5% for the lack of professional fulfilment), with an overall burnout prevalence of 58.2%.

Although a large number of factors demonstrated a significant association with burnout domains, few variables could significantly predict the high scores of burnout domains after controlling for other factors. These predictors included the young age of the residents (≤ 30 years), a high total number of hours worked per week (>80 h/week), lack of satisfaction or being neither satisfied nor dissatisfied with a career in medicine, dissatisfaction with efficiency and resources, and feeling that the residency program *somewhat* has enough strategies aimed at residents’ well-being in place (*p*< 0.05).

### 4.2. Burnout, Sociodemographic, and Professional Data

Burnout rates among residents in our study were consistent with the findings reported in the literature. Across multiple specialties, 30% to 74% of the residents reported burnout [1,45]. According to a recent systematic review that examined burnout among otolaryngology residents, the overall rates of burnout among residents ranged from 29.7% to 86% [46]. In our study, surgical specialties reported higher rates of burnout compared to other specialties, on almost all burnout domains. Consistent with this finding, high burnout rates have been reported among surgical residents in specialties such as orthopedic [1,45], obstetrics and gynecology [26,47], otolaryngology [46], and neurosurgery [27].

Regarding the demographic characteristics of the participating residents in our study, it was not surprising that, except for the age of residents, there was no statistically significant association between burnout outcome domains and the remaining demographic characteristics. This was demonstrated in four multivariate logistic regression models, after controlling for other variables. Likewise, based on two previous literature reviews examining burnout among residents [2,3], the authors reported that demographic factors are not reliably associated with burnout among residents; their conclusion was based on the replicability of this negative association. For example, the gender of the residents in our study was not significantly associated with burnout. However, in another article, while similar to our conclusion in most of its reviewed papers, the authors report other contradicting views, such as women physicians being more likely to report symptoms of burnout and feeling lack of efficacy while male physicians are less likely to doubt the quality of their work [6].

Regarding occupational stresses and personal life threats, in our study more than one in four residents reported their dissatisfaction with work–life integration (42.9%). Dissatisfaction with work–life balance is generally not uncommon among health professionals; in one study, physicians were twice as likely to be dissatisfied with their work–life balance (40.2%) compared to a control population of non-physicians (23.2%) [9]. Work–life balance refers to the amount of time spent doing one’s job compared with the amount of time spent with family and doing enjoyable things [48]. In the context of residency training, several factors have had a meaningful association with work–life imbalance such as having long working hours/days, few vacations, frequent calls, frequent days of working-as-usual after overnight surgery, perceived high-level job stress, and low satisfaction with human relationships [49].

### 4.3. Burnout Correlates and Comparisons

A closer look at the factors significantly associated with burnout among residents reveals that residents’ satisfaction with their career was inversely related to burnout and depression [26,47]. Our study found a strong association between residents’ satisfaction with their career and depersonalization and emotional exhaustion burnout domains, consistent with the findings of a study run on obstetrics and gynecology residents, where high depersonalization was correlated with low job satisfaction and personal accomplishment [47]. Furthermore, job dissatisfaction was closely related to experiencing depression symptoms among residents [26,47].

Additionally, the working hours factor was closely associated with burnout, where the residents in this study who worked more than 80 h per week were more likely to experience high depersonalization and high work exhaustion and interpersonal disengagement (sixteen and five times, respectively). In response to a survey sent to the residents of otolaryngology and head and neck surgery in the US, 684 responses were received and reported that the hours worked was the strongest associated factor predicting emotional exhaustion, with a rising emotional exhaustion score of 0.19 for each additional hour worked [28]. Similarly, total work hours were significantly correlated with reported stress, burnout, and less sleep, resulting in deleterious effects on residents’ performance in terms of fewer educational accomplishments, involvement in personal accident or injury, a severe conflict, or a significant medical error, particularly among residents who work more than 80 h per week [42,43,44].

In our study, feeling that the residency program somewhat has enough strategies aimed at resident well-being in place was associated with more than three times the likelihood of experiencing work exhaustion and interpersonal disengagement compared to those who agreed that the residency program has enough strategies for residents’ well-being. This finding represents a key message for policy and practice planners to appreciate the importance of well-being and support offered to the residents throughout their training. When the aim is to prevent and treat burnout, programs such as stress management, ranging from relaxation to cognitive-behavioral therapy, and patient-centered therapy were found to be of a strong significant impact [21].

As noted in our study, residents of younger age (≤30 years) experienced significantly more burnout symptoms (23 times) compared to older residents. We hardly found supportive research providing the same finding. Most researchers report the lack of association between demographic factors, including the age of residents, and burnout. Rather, the evident relationship was described in relation to occupational stressors, such as hostile faculty or co-residents, underappreciation by the patients, or poor control over one’s schedule [2,3]. However, individuals of a younger age generally seem to consistently report high levels of stress, anxiety, PTSD, and depression, when compared to older individuals, particularly during natural disasters, such as the COVID-19 pandemic [50,51,52,53].

Satisfaction with efficiency and resources was a significant predictor of burnout. Residents who were not satisfied with efficiency and resources were 11 times more likely to experience burnout, compared to those who were satisfied. This is not surprising, since the available resources represent an integral component of burnout development, where an imbalance between demand and resources may lead to strain [54]. Two theories of burnout development have been confirmed in research; the first model represents the Job Demands–Resources model. In this model, burnout arises when individuals experience increasing job demands while having inadequate resources to address and reduce these demands. The second model is the Conservation of Resources model, where burnout arises because of persistent threats to the available resources [54]. When individuals perceive that the resources they value are threatened or lost they strive to maintain those resources, which may aggravate burnout [54].

### 4.4. Limitations of the Study

This study has a number of limitations. The response rate was approximately 10% and thus fairly low. The projected sample size was 959 based upon a 95% confidence level and 2% margin of error for burnout prevalence; however, the study achieved a much smaller sample size. Thus, based on a population sample of 157, the actual margin of error was 7%, which is higher than the projected 2% determined a priori. Nonetheless, this low response rate puts this study at increased risk for sampling bias, specifically that the minority of residents who responded may be divergent in some way from the majority who did not respond, which could affect results. The low response rate can also potentially limit the external validity of the study, as the results may not be generalizable to the resident population at large. However, this low response rate was consistent with the other literature, including the rate reported in a national resident survey collecting data from the residents about their experience and opinion in residency training in Canada [55]. The survey achieved only an 8.3% response rate (833 residents completed the survey out of 10,091 residents).

Secondly, the study was cross-sectional in nature and therefore may not have predictive ability compared to a longitudinal study. It is also possible that the cross-sectional results may have been altered due to the ongoing COVID-19 global pandemic occurring during data collection. Lastly, the study depended on self-reported data collected from resident doctors regarding their sociodemographic, professional, support, and burnout information, lacking clinicians’ encounters to verify reported data and related mental health conditions.

## 5. Conclusions and Future Directives

Burnout is a serious phenomenon that can degenerate into mental health conditions or become disruptive to one’s professional performance, well-being and patient care. Lack of sleep and more working hours are well-known associated risks for increasing rates of depression, stress, alcohol use, suicidality, and committing medical errors among residents who have experienced burnout [1,18,19,20,21].

Our study confirmed high burnout levels (58.2%) among resident doctors in the province of Alberta in Canada. Based on the run of various multivariate binary logistic regression models in this study, some correlates demonstrated significant association with high burnout levels, including training-related factors, along with the age of residents. Some specialties showed more predilection to high rates of burnout, albeit insignificantly after controlling for other variables in the logistic regression models. The relationship between specialty and burnout could be better investigated in a larger, more representative sample. Such factors may need to be captured and addressed with reforming directives that aim to improve the residency training experience and achieve a better well-being profile of the residents.

A number of interventions have been proposed to mitigate burnout symptoms among residents, with promising outcomes. For example, duty-hour restrictions, self-development groups, training in mindfulness, formal trainee mentorship programs communication, and stress management, meaningful mentorship, self-development groups, the Respiratory One Method for relaxation, and conversion to a pass-fail grading system appear to reduce burnout [2,46,56]. To this end, leaders of medical schools, stakeholders and policy makers need to acknowledge, design, and implement various strategies capable of providing continuous and effective wellness support to residents. In addition, features of work such as workload, control and flexibility, and efficiency and resources represent areas that require systemic quality improvement that extends beyond mental health and wellness support for residents. Further research needs to be conducted not only on the prevalence and correlates of burnout among residents, but also on feasible effective support strategies aiming at improving the psychological health of resident doctors across Canada.

## Figures and Tables

**Table 1:** **A, B, C, D, and E.** Frequency distribution of socio-demographic, professional, support, and burnout characteristics of the respondents.

**(A)** **Sociodemographic and Academic Factors**	**Category**		**Frequency (%)**
Age (years)	<30		83 (59.3)
	>30		57 (40.7)
Gender	Male		55 (39.3)
	Female		85 (60.7)
Dependents	No		113 (72.0)
	Yes		44 (28.0)
Relationship Status	In a relationship		109 (77.9)
	Not in a Relationship		31 (22.1)
Ethnicity	Caucasian		92 (65.7)
	Others		48 (34.3)
How much debt do you currently have	<USD 100,000		73 (52.1)
	>USD 100,000		67 (47.9)
Year of residency training	PGY-1		36 (25.7)
	PGY-2		34 (24.3)
	PGY-3		19 (13.6)
	PGY-4		27 (19.3)
	PGY-5		14 (10.0)
	PGY-6		10 (7.1)
How many hours do you work per week	<80 h		104 (74.3)
	>80 h		53 (25.7)
What residency program are you part of	Surgical Specialties		26 (18.6)
	Family Medicine		33 (23.6)
	Internal Medicine		33 (23.6)
	Psychiatry		14 (10.0)
	Others		34 (24.3)
**(B)** **Professional Satisfaction**	**Satisfied** **N (%)**	**Neither Satisfied/Dissatisfied** **N (%)**	**Dissatisfied** **N (%)**
(1) Quality of peer collab. among resident colleagues	101 (75.9)	18 (13.5)	14 (10.5)
(2) Quality of interaction with attending physicians	102 (76.7)	17 (12.8)	14 (10.5)
(3) Quality of learning environment	92 (69.2)	23 (17.3)	18 (13.5)
(4) Workload and job demand	60 (45.1)	30 (22.6)	43 (32.3)
(5) Control and flexibility	35 (26.3)	27 (20.3)	71 (53.4)
(6) Work–life integration	40 (30.1)	36 (27.1)	57 (42.9)
(7) Efficiency and resources	47 (35.3)	51 (38.3)	35 (26.3)
**(C)** **Workplace Collegiality**	**Agree**	**Neither Agree nor Disagree**	**Disagree**
(1) Overall, I am satisfied with my career in medicine	80 (62.0)	31 (24.0)	18 (14.0)
(2) I find colleagues to be supportive	108 (83.7)	17 (13.2)	4 (3.1)
(3) People treat each other with respect in my work group	101 (78.3)	17 (13.2)	11 (8.5)
(4) A spirit of cooperation and teamwork exists in my group	101 (78.3)	16 (12.4)	12 (9.3)
(5) Disputes or conflicts are resolved fairly in my work groups	82 (63.6)	30 (23.3)	17 (13.2)
**(D)** **Support Variables**			
(1) How well do you feel supported by your social support/friends/family	Somewhat/Very well, N (%)	Neutral, N (%)	Somewhat/Very poorly, N (%)
	105 (87.5)	8 (6.7)	7 (5.8)
(2) How would you describe what would occur if you reached out for help to those in your learning and work environment	Somewhat/very supportive	Neutral	Somewhat/very hostile
	94 (78.3)	14 (11.7)	12 (10.0)
(3) Do you feel that your residency program has enough strategies aimed at residents’ well-being in place	Yes	Somewhat	No
	38 (31.7)	46 (38.3)	36 (30.0)
**(E)** **Burnout Variables**	**Category**		**Frequency (%)**
(1) Depersonalization	Absent		77 (59.7)
	Present		52 (40.3)
(2) Emotional exhaustion	Absent		67 (51.9)
	Present		62 (48.1)
(3) Work exhaustion and interpersonal disengagement	Absent		55 (43.3)
	Present		72 (56.7)
(4) Professional fulfillment scale	Absent		106 (83.5)
	Present		21 (16.5)

**Table 2:** Association between demographic, professional and workplace variables, and depersonalization and emotional exhaustion.

Characteristic	Depersonalization			Emotional Exhaustion		
	(Present)			(Present)		
	N (%)	*p*-Value	Effect Size	N (%)	*p*-Value	Effect Size
			Phi/Cramer V *			Phi/Cramer V *
Age						
<30	31 (40.8)	0.894	0.012	37 (48.7)	0.999	0.015
>30	21 (39.6)			25 (47.2)		
Gender						
Male	23 (46.0)	0.358	0.092	26 (52.0)	0.588	0.063
Female	29 (36.7)			36 (45.6)		
Dependent						
No	34 (38.6)	0.57	0.05	46 (52.3)	0.188s	0.123
Yes	18 (43.9)			16 (39.0)		
Relationship						
In a relationship	39 (38.6)	0.516	0.066	47 (46.5)	0.529	0.058
Not in a relationship	13 (46.4)			15 (53.6)		
Ethnicity						
Caucasian	35 (41.2)	0.851	0.025	42 (49.4)	0.713	0.038
Other	17 (38.6)			20 (45.5)		
How much debt do you currently have						
≤USD 100,000	27 (40.9)	0.999	0.012	32 (48.5)	0.999	0.009
>USD 100,000	25 (39.7)			30 (47.6)		
What year of residency training are you currently in?						
PGY-1	14 (38.9)	0.352	0.209	18 (50.0)	0.452	0.193
PGY-2	9 (30.0)			11 (36.7)		
PGY-3	10 (58.8)			11 (64.7)		
PGY-4	12 (48.0)			12 (48.0)		
PGY-5	3 (25.0)			7 (58.3)		
PGY-6 and above	4 (44.4)			3 (33.3)		
What residency program are you part of						
Surgical Specialties	16 (64.0)	0.041 *	0.278	14 (56.0)	0.678	0.135
Family Medicine	8 (25.8)			13 (41.9)		
Internal Medicine	11 (37.9)			15 (51.7)		
Psychiatry	3 (25.0)			4 (33.3)		
Others	14 (43.8)			16 (50.0)		
How many total hours do you work per week (clinicals and non-clinicals)						
<80 h	27 (28.4)	<0.001 *	0.405	37 (38.9)	<0.001 *	0.305
>80 h	25 (73.5)			25 (73.5)		
Quality of peer collaboration among residents’ colleagues						
Satisfied	36 (37.1)	0.355	0.13	42 (43.3)	0.001	0.314
Neither	8 (44.4)			7 (38.9)		
Dissatisfied	8 (57.1)			13 (92.9)		
Quality of interaction with your attending physicians						
Satisfied	34 (34.7)	0.029 *	0.237	40 (40.8)	0.013 *	0.258
Neither	8 (47.1)			12 (70.6)		
Dissatisfied	10 (71.4)			10 (71.4)		
Quality of your learning environment						
Satisfied	28 (31.1)	<0.001 *	0.364	33 (36.7)	<0.001 *	0.402
Neither	9 (42.9)			12 (57.1)		
Dissatisfied	15 (83.3)			17 (94.4)		
Workload and job demand						
Satisfied	13 (22.8)	<0.001 *	0.389	18 (31.6)	<0.001 *	0.397
Neither	11 (36.7)			12 (40.0)		
Dissatisfied	28 (66.7)			32 (76.2)		
Control and flexibility						
Satisfied	6 (18.2)	0.012 *	0.264	10 (30.3)	0.002 *	0.308
Neither	12 (48.0)			8 (32.0)		
Dissatisfied	34 (47.9)			44 (62.0)		
Work–life integration (meeting personal and professional obligations)						
Satisfied	8 (21.1)	<0.001 *	0.398	12 (31.6)	<0.001 *	0.378
Neither	9 (25.7)			11 (31.4)		
Dissatisfied	35 (62.5)			39 (69.6)		
Efficiency and resources						
Satisfied	11 (25.0)	0.003 *	0.302	13 (29.5)	<0.001 *	0.404
Neither	19 (38.0)			21 (42.0)		
Dissatisfied	22 (62.9)			28 (80.0)		
Overall, I am satisfied about pursuing a career in medicine						
Agree	15 (18.8)	<0.001 *	0.562	24 (30.0)	<0.001	0.488
Neither agree/disagree	23 (74.2)			21 (67.7)		
Disagree	14 (77.8)			17 (94.4)		
In general, I find my colleagues to be supportive						
Agree	36 (33.3)	<0.001 **	0.323	43 (39.8)	<0.001 **	0.376
Neither agree/disagree	13 (76.5)			15 (88.2)		
Disagree	3 (75.0)			4 (100.0)		
People treat each other with respect in my work group						
Agree	37 (36.6)	0.167	0.162	45 (44.6)	0.198	0.161
Neither agree/disagree	8 (47.1)			9 (52.9)		
Disagree	7 (63.6)			8 (72.7)		
A spirit of cooperation and teamwork exists in my work group						
Agree	39 (38.6)	0.456	0.118	43 (42.6)	0.048 *	0.216
Neither agree/disagree	6 (37.5)			10 (62.5)		
Disagree	7 (58.3)			9 (75.0)		
Disputes or conflicts are resolved fairly in my work group						
Agree	23 (28.0)	<0.001 *	0.357	32 (39.0)	0.001 *	0.326
Neither agree/disagree	16 (53.3)			15 (50.0)		
Disagree	13 (76.5)			15 (88.2)		
How well do you feel supported by your social support/friends/family?						
Somewhat/very well	38 (36.2)	0.023 **	0.249	47 (44.8)	0.382 **	0.126
Neutral	2 (25.0)			4 (50.0)		
Somewhat/very poorly	6 (85.7)			5 (71.4)		
How would you best describe what would occur if you reached out for help to those in your learning and work environment						
Somewhat/very supportive	30 (31.9)	0.008 *	0.278	37 (39.4)		
Neutral	7 (50.0)			10 (71.4)	0.009 *	0.279
Somewhat/very hostile	9 (75.0)			9 (75.0)		
Do you feel that your residency has enough strategies aimed at residents’ well-being in place?						
Yes	8 (21.1)	0.004 *	0.302	11 (28.9)		
Somewhat	17 (37.0)			22 (47.8)	0.011 *	0.276
No	21 (58.3)			23 (63.9)		

* *p* value < 0.05, ** fisher exact.

**Table 3:** Association between demographic, professional and workplace variables and work exhaustion and interpersonal disengagement and professional fulfilment.

Characteristic	Work Exhaustion and Interpersonal Disengagement			Professional Fulfilment		
	(Present)			(Absent)		
	N (%)	*p*-Value	Effect Size	N (%)	*p*-Value	Effect Size
			Phi/Cramer V *			Phi/Cramer V *
Age						
<30	44 (58.7)	0.716	0.048	67 (89.3)	0.050 *	0.19
>30	28 (53.8)			39 (75.0)		
Gender						
Male	29 (60.4)	0.581	0.059	39 (81.3)	0.628	0.046
Female	43 (54.4)			67 (84.8)		
Dependent						
No	51 (58.6)	0.56	0.057	74 (85.1)	0.608	0.063
Yes	21 (52.2)			32 (80.0)		
Relationship						
In a relationship	56 (56.6)	0.999	0.005	80 (80.8)	0.159 *	0.134
Not in a relationship	16 (57.1)			26 (92.9)		
Ethnicity						
Caucasian	46 (54.8)	0.575	0.054	72 (85.7)	0.449	0.085
Others	26 (60.5)			34 (79.1)		
How much debt do you currently have						
<USD 100,000	35 (53.8)	0.592	0.059	54 (83.1)	0.999	0.011
>USD 100,000	37 (59.7)			52 (83.9)		
What year of residency training are you currently in?						
PGY-1	22 (64.7)	0.351	0.211	27 (79.4)	0.342 **	0.195
PGY-2	12 (40.0)			24 (80.0)		
PGY-3	12 (70.6)			17 (100.0)		
PGY-4	14 (56.0)			21 (84.0)		
PGY-5	7 (58.3)			9 (75.0)		
PGY-6 and above	5 (55.6)			8 (88.9)		
What residency program are you part of						
Surgical Specialties	16 (69.6)	0.207	0.217	23 (100.0)	0.060 **	0.247
Family Medicine	13 (41.9)			23 (74.2)		
Internal Medicine	16 (55.2)			24 (82.8)		
Psychiatry	9 (75.0)			11 (91.7)		
Others	18 (56.3)			25 (78.1)		
How many total hours do you work per week (clinicals and non-clinicals)						
<80 h	45 (47.9)	0.001 *	0.3	76 (80.9)	0.276	0.119
>80 h	27 (81.8)			30 (90.9)		
Quality of peer collaboration among residents’ colleagues						
Satisfied	50 (51.5)	0.108	0.189	78 (80.4)	0.203 **	0.166
Neither	12 (70.6)			15 (88.2)		
Dissatisfied	10 (76.9)			13 (100.0)		
Quality of interaction with your attending physicians						
Satisfied	48 (49.5)	0.008 *	0.267	77 (79.4)	0.079 **	0.203
Neither	12 (75.0)			16 (100.0)		
Dissatisfied	12 (85.7)			13 (92.9)		
Quality of your learning environment						
Satisfied	43 (47.8)	0.003 *	0.302	70 (77.8)	0.018 **	0.242
Neither	13 (68.4)			18 (94.7)		
Dissatisfied	16 (88.9)			18 (100.0)		
Workload and job demand						
Satisfied	23 (40.4)	<0.001 *	395	40 (70.2)	0.001 *	0.331
Neither	15 (50.0)			27 (90.0		
Dissatisfied	34 (85.0)			39 (97.5)		
Control and flexibility						
Satisfied	8 (24.2)	<0.001 *	0.441	18 (54.5)	<0.001 *	0.468
Neither	12 (48.0)			22 (88.0)		
Dissatisfied	52 (75.4)			66 (95.7)		
Work–life integration (meeting personal and professional obligations)						
Satisfied	15 (39.5)	0.001 *	0.337	25 (65.8)	<0.001 *	0.37
Neither	16 (45.7)			28 (80.0)		
Dissatisfied	41 (75.9)			53 (98.1)		
Efficiency and resources						
Satisfied	12 (27.3)	<0.001 *	0.436	27 (61.4)	<0.001 *	0.434
Neither	34 (69.4)			46 (93.9)		
Dissatisfied	26 (76.5)			33 (97.1)		
Overall, I am satisfied about pursuing a career in medicine						
Agree	35 (43.8)	<0.001 *	0.342	60 (75.0)	0.002 **	0.298
Neither agree/disagree	23 (76.7)			29 (96.7)		
Disagree	14 (82.4)			17 (100.0)		
In general, I find my colleagues to be supportive						
Agree	56 (52.3)	0.076 **	0.204	87 (81.3)		
Neither agree/disagree	13 (81.3)			15 (93.8)	0.455 **	0.137
Disagree	3 (75.0)			4 (100.0)		
People treat each other with respect in my work group						
Agree	51 (51.0)	0.042 *	0.223	80 (80.0)	0.107 **	0.188
Neither agree/disagree	12 (75.0)			16 (100.0)		
Disagree	9 (81.8)			10 (90.9)		
A spirit of cooperation and teamwork exists in my work group						
Agree	52 (52.5)	0.2	0.161	80 (80.8)	0.432 **	0.135
Neither agree/disagree	11 (68.8)			15 (93.8)		
Disagree	9 (75.0)			11 (91.7)		
Disputes or conflicts are resolved fairly in my work group						
Agree	36 (45.0)	0.002 *	0.312	61 (76.3)	0.013 **	0.254
Neither agree/disagree	22 (73.3)			29 (96.7)		
Disagree	14 (82.4)			16 (94.1)		
How well do you feel supported by your social support/friends/family?						
Somewhat/very well	58 (55.2)	0.919 **	0.037	86 (81.9)	0.739 **	0.117
Neutral	5 (62.5)			7 (87.5)		
Somewhat/very poorly	4 (57.1)			7 (100.0)		
How would you best describe what would occur if you reached out for help to those in your learning and work environment						
Somewhat/very supportive	49 (52.1)	0.177	0.171	75 (79.8)	0.120 **	0.188
Neutral	11 (78.6)			14 (100.0)		
Somewhat/very hostile	7 (58.3)			11 (91.7)		
Do you feel that your residency program has enough strategies aimed at residents’ well-being in place						
Yes	9 (23.7)	<0.001 *	0.441	24 (63.2)		
Somewhat	33 (71.7)			41 (89.1)	<0.001 *	0.379
No	25 (69.4)			35 (97.2)		

* significance < 0.05, ** Fisher’s Exact Test was used.

**Table 4:** Summary of the significant results of the logistic regression analysis models predicting the likelihood of depersonalization, emotional exhaustion, work exhaustion and interpersonal disengagement, and professional fulfilment.

Characteristics	(1) High Depersonalization			(2) High Emotional Exhaustion			(3) Professional Fulfilment Index					
							a. **High Work Exhaustion and Interpersonal Disengagement**			b. **Low Professional Fulfilment**		
	OR	95% CI	*p*-Value	OR	95% CI	*p*-Value	OR	95% CI	*p*-Value	OR	95% CI	*p*-Value
How many total hours do you work per week (clinical and non-clinical) >80 h	16.43	2.06–131.23	0.008	_	_	_	5.365	1.09–26.42	0.04	_	_	_
Overall, I am satisfied with my career in medicine Agree Neither agree/disagree Disagree	23.82 22.29	4.90–115.87 1.75–283.28	<0.001 <0.001 0.02	5.14 10.88	1.325–19.94 0.269–440.52	0.04 0.02 0.21	_	_	_	---	--	--
Efficiency and resources Satisfied Neither Dissatisfied	_	--	_	1.33 10.83	0.35–5.04 1.67–70.32	0.04 0.68 0.01	_	--	_	--	--	--
Do you feel that your residency program has enough strategies aimed at residents’ well-being in place? Yes Somewhat No	--	--	--	--	--	--	3.70 0.65	1.10–12.46 0.112–3.82	0.03 0.04 0.64	--	--	--
Age >30 years	--	--	--	--	--	--	--	--	--	0.04	0.005–0.298	0.002

## Data Availability

The data that support the findings of this study are available from the corresponding author, Vincent Agyapong, upon reasonable request.

## Appendix A

**Table A1:** Logistic regression predicting the likelihood of Depersonalization.

Variables in Equation	B	S.E.	Wald	df	Sig.	Odd’s Ratio	95% C.I. for Odd’s Ratio	
							Lower	Upper
What residency program are you a part of?								
Surgical Specialties			2.853	4	0.583			
Family Medicine	1.512	1.216	1.545	1	0.214	4.536	0.418	49.224
Internal Medicine	0.075	1.075	0.005	1	0.944	1.078	0.131	8.862
Psychiatry	0.603	1.634	0.136	1	0.712	1.828	0.074	44.945
Others	1.287	1.139	1.278	1	0.258	3.623	0.389	33.746
How many total hours do you work per week (clinical and non-clinical)								
>80	2.8	1.06	6.977	1	0.008	16.43	2.059	131.225
Quality of interaction with your attending physicians								
Satisfied			1.397	2	0.497	7		
Neither	−0.948	1.048	0.818	1	0.366	0.388	0.05	3.024
Dissatisfied	−1.877	1.753	1.146	1	0.284	0.153	0.005	4.754
Quality of your learning environment								
Satisfied			1.586	2	0.452			
Neither	0.754	1.043	0.523	1	0.47	2.126	0.275	16.427
Dissatisfied	2.078	1.654	1.578	1	0.209	7.989	0.312	204.4
Workload and job demands								
Satisfied			0.034	2	0.983			
Neither	−0.122	0.879	0.019	1	0.89	0.885	0.158	4.957
Dissatisfied	0.028	1.226	0.001	1	0.982	1.028	0.093	11.361
Control and flexibility								
Satisfied			2.2	2	0.333			
Neither	1.04	0.896	1.347	1	0.246	2.828	0.489	16.371
Dissatisfied	−0.084	1.016	0.007	1	0.934	0.919	0.125	6.74
Efficiency and resources								
Satisfied			0.379	2	0.827			
Neither	−0.118	0.779	0.023	1	0.879	0.889	0.193	4.091
Dissatisfied	0.387	0.965	0.161	1	0.688	1.473	0.222	9.771
Overall, I am satisfied with my career in medicine								
Agree			17.585	2	<0.001			
Neither agree/disagree	3.17	0.807	15.428	1	<0.001	23.817	4.896	115.865
Disagree	3.104	1.297	5.726	1	0.017	22.289	1.754	283.278
In general, I find my colleagues to be supportive								
Agree			0.486	2	0.784			
Neither agree/disagree	−0.225	1.102	0.042	1	0.838	0.799	0.092	6.924
Disagree	−1.186	1.708	0.482	1	0.487	0.305	0.011	8.687
Disputes or conflicts are resolved fairly in my work group								
Agree			0.881	2	0.644			
Neither agree/disagree	0.444	0.805	0.304	1	0.581	1.559	0.322	7.559
Disagree	1.214	1.327	0.837	1	0.36	3.367	0.25	45.373
How well do you feel supported by your social supports/friends/family?								
Somewhat/very well			1.664	2	0.435			
Neutral	0.142	1.286	0.012	1	0.912	1.152	0.093	14.331
Somewhat/very poorly	2.082	1.624	1.644	1	0.2	8.024	0.332	193.671
How would you best describe what would occur if you reached out for help to those in your learning and work environment?								
Somewhat/very supportive			0.801	2	0.67			
Neutral	−0.985	1.1	0.801	1	0.371	0.374	0.043	3.228
Somewhat/very hostile	−0.606	1.367	0.196	1	0.658	0.546	0.037	7.96
Do you feel that your residency program has enough strategies aimed at residents’ well-being in place?								
Yes			0.005	2	0.998			
Somewhat	−0.003	0.793	0	1	0.997	0.997	0.211	4.712
No	0.057	1.038	0.003	1	0.956	1.059	0.138	8.102
Constant	−3.626	1.269	8.168	1	0.004	0.027		

**Table A2:** Logistic regression predicting the likelihood of Emotional Exhaustion.

Variables in Equation	B	S.E.	Wald	df	Sig	Odd’s Ratio	95% C.I. for Odd’s Ratio	
							Lower	Upper
How many total hours do you work per week (clinical and non-clinical) >80	0.854	0.891	0.918	1	0.338	2.348	0.410	13.465
Quality of peer collaboration among your resident colleagues Satisfied Neither Dissatisfied	−0.903 19.109	1.266 5044.959	0.509 0.509 0.000	2 1 1	0.775 0.476 0.997	0.405 1989955180.044	0.034 0.000	4.844
Quality of interaction with your attending physicians Satisfied Neither Dissatisfied	−0.062 −28.782	1.238 9505.315	0.002 0.002 0.000	2 1 1	0.999 0.960 0.998	0.940 0.000	0.083 0.000	10.63
Quality of your learning environment Satisfied Neither Dissatisfied	1.319 31.582	1.010 9505.315	1.705 1.705 0.000	2 1 1	0.426 0.192 0.997	3.740 51969774812758.520	0.516 0.000	27.08
Control and flexibility Satisfied Neither Dissatisfied	0.090 0.497	0.864 0.864	0.395 0.011 0.331	2 1 1	0.821 0.917 0.565	1.094 1.644	0.201 0.302	5.949 8.945
Work–life integration (meeting personal and professional obligations) Satisfied Neither Dissatisfied	−0.145 −0.829	0.731 0.923	0.826 0.040 0.807	2 1 1	0.662 0.842 0.369	0.865 0.436	0.206 0.072	3.626 2.664
Efficiency and resources Satisfied Neither Dissatisfied	0.285 2.382	0.679 0.955	6.541 0.176 6.225	2 1 1	0.038 0.675 0.013	1.329 10.825	0.351 1.666	5.035 70.322
Overall, I am satisfied with my career in medicine Agree Neither agree/disagree Disagree	1.637 2.387	0.692 1.888	6.391 5.601 1.598	2 1 1	0.041 0.018 0.206	5.140 10.879	1.325 0.269	19.939 440.516
In general, I find my colleagues to be supportive Agree Neither agree/disagree Disagree	52.447 56.062	9498.519 15311.532	0.000 0.000 0.000	2 1 1	1.000 0.996 0.997	59917769681138890000000.000 2225162605634998200000000.000	0.000 0.000	
A spirit of cooperation and teamwork exists in my work group Agree Neither agree/disagree Disagree	0.844 −36.107	1.342 8030.410	0.395 0.395 0.000	2 1 1	0.821 0.530 0.996	2.325 0.000	0.168 0.000	32.280
Disputes or conflicts are resolved fairly in my work group Agree Neither agree/disagree Disagree	0.202 −1.498	0.844 3.903	0.262 0.057 0.147	2 1 1	0.877 0.811 0.701	1.224 0.224	0.234 0.000	6.406 469.623
How would you best describe what would occur if you reached out for help to those in your learning and work environment? Somewhat/very supportive Neutral Somewhat/very hostile	0.248 −2.291	1.153 1.765	2.027 0.046 1.684	2 1 1	0.363 0.830 0.194	1.282 0.101	0.134 0.003	12.281 3.220
Do you feel that your residency program has enough strategies aimed at resident well-being in place? Yes Somewhat No	−0.625 0.695 0.991	−0.474	0.612 0.466 0.397	2 1 1	0.736 0.495 0.529	0.622 0.535	0.159 0.077	2.431 3.737
Constant	−1.546	0.587	6.927	1	0.008	0.213		

**Table A3:** Logistic regression predicting the likelihood of Work Exhaustion and Interpersonal Disengagement.

Variables in Equation	B	S.E.	Wald	df	Sig.	Odd’s Ratio	95% C.I. for Odd’s Ratio	
							Lower	Upper
How many total hours do you work per week (clinical and non-clinical) >80	1.680	0.813	4.265	1	0.039	5.365	1.089	26.418
Quality of interaction with your attending physicians Satisfied Neither Dissatisfied	0.862 0.547	0.917 1.294	0.892 0.885 0.179	2 1 1	0.640 0.347 0.672	2.369 1.728	0.393 0.137	14.288 21.807
Quality of your learning environment Satisfied Neither Dissatisfied	0.396 1.035	0.878 1.489	0.524 0.204 .483	2 1 1	0.770 0.652 .487	1.486 2.815	0.266 .152	8.301 52.087
Workload and job demands Satisfied Neither Dissatisfied	−1.050 0.012	0.677 0.961	3.083 2.405 0.000	2 1 1	0.214 0.121 0.990	0.350 1.012	0.093 0.154	1.319 6.663
Control and flexibility Satisfied Neither Dissatisfied	−0.218 1.195	0.783 0.755	4.707 0.078 2.507	2 1 1	0.095 0.780 0.113	0.804 3.303	0.173 0.753	3.728 14.498
Efficiency and resources Satisfied Neither Dissatisfied	1.309 1.014	0.621 0.783	4.521 4.445 1.674	2 1 1	0.104 0.035 0.196	3.702 2.756	1.097 0.593	12.497 12.799
Overall, I am satisfied with my career in medicine Agree Neither agree/disagree Disagree	1.268 0.339	0.684 1.257	3.462 3.437 0.073	2 1 1	0.177 0.064 0.788	3.555 1.403	0.930 0.119	13.589 16.482
In general, I find my colleagues to be supportive Agree Neither agree/disagree Disagree	−0.315 −3.504	1.103 2.479	2.025 0.082 1.997	2 1 1	0.363 0.775 0.158	0.730 0.030	0.084 0.000	6.342 3.879
People treat each other with respect in my work group Agree Neither agree/disagree Disagree	−1.273 0.919	.924 1.755	2.696 1.895 .274	2 1 1	0.260 0.169 0.600	0.280 2.507	0.046 0.080	1.714 78.116
Disputes or conflicts are resolved fairly in my work group Agree Neither agree/disagree Disagree	1.028 0.822	0.738 1.292	1.941 1.941 0.405	2 1 1	0.379 0.164 0.524	2.796 2.276	0.658 0.181	11.878 28.637
Do you feel that your residency program has enough strategies aimed at residents’ well-being in place? Yes Somewhat No	1.308 −0.424	0.619 0.901	7.170 4.461 0.222	2 1 1	0.028 0.035 0.638	3.699 0.654	1.099 0.112	12.455 3.824
Constant	−2.216	0.581	14.529	1	<0.001	0.109		

**Table A4:** D: Logistic regression predicting the likelihood of Professional Fulfilment.

Variables in Equation	B	S.E.	Wald	df	Sig.	Odd’s Ratio	95% C.I. for Odd’s Ratio	
							Lower	Upper
Age >30y	−3.131	1.184	6.993	1	0.008	0.044	0.004	0.445
What residency program are you a part of? Surgical Specialties Family Medicine Internal Medicine Psychiatry Others	−17.238 −18.007 −16.657 −17.901	6311.334 6311.334 6311.334 6311.334	0.83 0 0 0 0	4 1 1 1 1	0.934 0.998 0.998 0.998 0.998	0 0 0 0	0 0 0 0	
Quality of interaction with your attending physicians Satisfied Neither Dissatisfied	15.368 −21.125	6707.523 6297.332	0 0 0	2 1 1	1 0.998 0.997	4725319 0	0 0	
Quality of your learning environment Satisfied Neither Dissatisfied	17.604 34.854	6297.33 9127.446	0 0 0	2 1 1	1 0.998 0.997	44200648 1.37E+15	0 0	
Workload and job demands Satisfied Neither Dissatisfied	−0.944 −0.749	1.171 1.999	0.668 0.65 0.14	2 1 1	0.716 0.42 0.708	0.389 0.473	0.039 0.009	3.86 23.796
Control and flexibility Satisfied Neither Dissatisfied	2.045 2.52	1.155 1.449	4.525 3.136 3.024	2 1 1	0.104 0.077 0.082	7.73 12.431	0.804 0.726	74.355 212.848
Efficiency and resources Satisfied Neither Dissatisfied	1.249 2.29	1.06 1.824	2.74 1.389 1.577	2 1 1	0.254 0.239 0.209	3.488 9.875	0.437 0.277	27.849 352.29
Overall, I am satisfied with my career in medicine Agree Neither agree/disagree Disagree	2.125 15.841	1.478 6280.807	2.066 2.066 0	2 1 1	0.356 0.151 0.998	8.37 7582288	0.462 0	151.7
Disputes or conflicts are resolved fairly in my work group Agree Neither agree/disagree Disagree	1.266 −2.082	1.62 5.298	0.793 0.611 0.154	2 1 1	0.673 0.435 0.694	3.547 0.125	0.148 0	84.921 4031.613
Do you feel that your residency program has enough strategies aimed at resident well-being in place? Yes Somewhat No	1.1 5.334	0.956 5.426	2.04 1.326 0.967	2 1 1	0.361 0.25 0.326	3.005 207.271	0.462 0.005	19.555 8607571
Constant	17.621	6311.334	0	1	0.998	44,931,092		

## References

[1] Ames S.E., Cowan J.B., Kenter K., Emery S., Halsey D. (2017). Burnout in Orthopaedic Surgeons: A Challenge for Leaders, Learners, and Colleagues: AOA Critical Issues. J. Bone Joint Surg. Am..

[2] Attenello F.J., Buchanan I.A., Wen T., Donoho D.A., McCartney S., Cen S.Y., Khalessi A.A., Cohen-Gadol A.A., Cheng J.S., Mack W.J. (2018). Factors associated with burnout among US neurosurgery residents: A nationwide survey. J. Neurosurg..

[3] Thomas N.K. (2004). Resident Burnout. JAMA.

[4] The L. (2021). Medical professionalism and physician wellbeing. Lancet.

[5] IsHak W.W., Lederer S., Mandili C., Nikravesh R., Seligman L., Vasa M., Ogunyemi D., Bernstein C.A. (2009). Burnout during residency training: A literature review. J. Grad. Med. Educ..

[6] De Hert S. (2020). Burnout in Healthcare Workers: Prevalence, Impact and Preventative Strategies. Local Reg. Anesth..

[7] Maslach C., Jackson S.E., Leiter M. (1996). Maslach Burnout Inventory Manual.

[8] Klimo P., DeCuypere M., Ragel B.T., McCartney S., Couldwell W.T., Boop F.A. (2013). Career satisfaction and burnout among U.S. neurosurgeons: A feasibility and pilot study. World Neurosurg..

[9] Shanafelt T.D., Boone S., Tan L., Dyrbye L.N., Sotile W., Satele D., West C.P., Sloan J., Oreskovich M.R. (2012). Burnout and satisfaction with work-life balance among US physicians relative to the general US population. Arch Intern. Med..

[10] Yang S., Meredith P., Khan A. (2015). Stress and burnout among healthcare professionals working in a mental health setting in Singapore. Asian J. Psychiatry.

[11] Dyrbye L., Shanafelt T. (2016). A narrative review on burnout experienced by medical students and residents. Med. Educ..

[12] Dyrbye L.N., West C.P., Satele D., Boone S., Tan L., Sloan J., Shanafelt T.D. (2014). Burnout among U.S. medical students, residents, and early career physicians relative to the general U.S. population. Acad. Med..

[13] (2020). Medscape National Physician Burnout & Suicide Report. https://www.medscape.com/slideshow/2020-lifestyle-burnout-6012460.

[14] (2020). Medscape Residents Lifestyle & Happiness Report. https://www.medscape.com/slideshow/2020-residents-lifestyle-happiness-6013110#2.

[15] (2021). Medscape Residents Lifestyle & Happiness Report. https://www.medscape.com/slideshow/2021-residents-lifestyle-happiness-6014240?_ga=2.17035641,1331399521,1662841552-1011102148,1656972621#2.

[16] Chang J., Ray J.M., Joseph D., Evans L.V., Joseph M. (2022). Burnout and Post-traumatic Stress Disorder Symptoms Among Emergency Medicine Resident Physicians During the COVID-19 Pandemic. West J. Emerg. Med..

[17] Canadian Physicians Report Declines in Mental Health, Professional Fulfillment. https://www.medscape.com/viewarticle/980260.

[18] West C.P., Shanafelt T.D., Kolars J.C. (2011). Quality of life, burnout, educational debt, and medical knowledge among internal medicine residents. JAMA.

[19] Kwok C. (2021). Depression, Stress, and Perceived Medical Errors in Singapore Psychiatry Residents. Acad. Psychiatry.

[20] Dyrbye L.N., Massie F.S., Eacker A., Harper W., Power D., Durning S.J., Thomas M.R., Moutier C., Satele D., Sloan J. (2010). Relationship between burnout and professional conduct and attitudes among US medical students. JAMA.

[21] Romani M., Ashkar K. (2014). Burnout among physicians. Libyan J. Med..

[22] Levinson W., Roter D. (1995). Physicians’ psychosocial beliefs correlate with their patient communication skills. J. Gen. Intern. Med..

[23] Suchman A.L., Roter D., Green M., Lipkin M. (1993). Physician satisfaction with primary care office visits. Collaborative Study Group of the American Academy on Physician and Patient. Med. Care.

[24] Hojat M., Louis D.Z., Markham F.W., Wender R., Rabinowitz C., Gonnella J.S. (2011). Physicians’ empathy and clinical outcomes for diabetic patients. Acad. Med..

[25] Shanafelt T.D., Bradley K.A., Wipf J.E., Back A.L. (2002). Burnout and self-reported patient care in an internal medicine residency program. Ann. Intern. Med..

[26] Becker J.L., Milad M., Klock S. (2006). Burnout, depression, and career satisfaction: Cross-sectional study of obstetrics and gynecology residents. Am. J. Obstet. Gynecol..

[27] Berardo L., Gerges C., Wright J., Stout A., Shah H., Papanastassiou A., Kimmell K. (2020). Assessment of burnout prevention and wellness programs for US-based neurosurgical faculty and residents: A systematic review of the literature. J. Neurosurg..

[28] Golub J.S., Weiss P.S., Ramesh A.K., Ossoff R.H., Johns M.M. (2007). Burnout in residents of otolaryngology-head and neck surgery: A national inquiry into the health of residency training. Acad. Med..

[29] Tang O.Y., Dunn K.A., Yoon J.S., Ponce F.A., Sonntag V.K., Lawton M.T. (2020). Neurosurgery Resident Wellness and Recovery from Burnout: A 39-Year Single-Institution Experience. World Neurosurg..

[30] Statistics Canada Canada at a Glance. Minister of Industry: Statistics Canada; CANSIM table 051-0005. 27 March 2018..

[31] Kim E., Mallett R., Hrabok M., Yang Y.A., Moreau C., Nwachukwu I., Kravtsenyuk M., Abba-Aji A., Li D., I O Agyapong V. (2020). Reducing Burnout and Promoting Health and Wellness Among Medical Students, Residents, and Physicians in Alberta: Protocol for a Cross-Sectional Questionnaire Study. JMIR Res. Protoc..

[32] CPSA Registeration Statistics. College of Physicians and Surgeons of Alberta; 2021–2017. https://cpsa.ca/wp-content/uploads/2022/01/Registration-Statistics-2021-2017,pdf.

[33] Overview of Utility of Mini Z. https://www.vumc.org/health-wellness/sites/vumc.org.health-wellness/files/public_files/PDFs/wlc/wlcMiniZ.pdf.

[34] Trockel M., Bohman B., Lesure E., Hamidi M.S., Welle D., Roberts L., Shanafelt T. (2018). A Brief Instrument to Assess Both Burnout and Professional Fulfillment in Physicians: Reliability and Validity, Including Correlation with Self-Reported Medical Errors, in a Sample of Resident and Practicing Physicians. Acad. Psychiatry.

[35] Canadian Medical Association (2018). CMA National Physician Health Survey: A National Snapshot October. https://www.cma.ca/sites/default/files/2018-11/nph-survey-e.pdf.

[36] Linzer M., Smith C.D., Hingle S., Poplau S., Miranda R., Freese R., Palamara K. (2020). Evaluation of Work Satisfaction, Stress, and Burnout Among US Internal Medicine Physicians and Trainees. JAMA Netw. Open.

[37] Professional Fulfillment & Burnout (PFI) McGill, Faculty of Medicine and Health Sciences. https://www.mcgill.ca/thewelloffice/pgme/wellness-support/self-screening-tools/professional-fulfillment-burnout-pfi.

[38] National Academy of Medicine Valid and Reliable Survey Instruments to Measure Burnout, Well-Being, and Other Work-Related Dimensions. https://nam.edu/valid-reliable-survey-instruments-measure-burnout-well-work-related-dimensions/.

[39] Complete Dissertaion by Statistics Solutions Maslach Burnout Inventory (MBI). https://www.statisticssolutions.com/free-resources/directory-of-survey-instruments/maslach-burnout-inventory-mbi/#:~:text=Reliability%20and%20Validity&text=Cronbach%20alpha%20ratings%20of%200.90,used%20for%20test%2Dretest%20reliability.

[40] Researchers to Study Severity & Impacts of Burnout on Alberta Physicians, Residents and Medical Students (2019). University of Alberta. https://www.ualberta.ca/psychiatry/news-and-events/news/2019/march/physician-burnout.html.

[41] IBM Corp (2020). Released 2019, IBM SPSS Statistics for Windows, Version 26.0..

[42] Baldwin D.C., Daugherty S.R., Tsai R., Scotti M.J. (2003). A national survey of residents’ self-reported work hours: Thinking beyond specialty. Acad. Med..

[43] Bui A.H., Ripp J.A., Oh K.Y., Basloe F., Hassan D., Akhtar S., Leitman I.M. (2020). The impact of program-driven wellness initiatives on burnout and depression among surgical trainees. Am. J. Surg..

[44] Larson D.P., Carlson M.L., Lohse C.M., O’Brien E.K., Kircher M.L., Gurgel R.K., Hunter J.B., Micco A.G., Nogan S.J., O’Connell B.P. (2021). Prevalence of and Associations With Distress and Professional Burnout Among Otolaryngologists: Part I, Trainees. Otolaryngol. Head Neck Surg..

[45] Arora M., Diwan A.D., Harris I.A. (2013). Burnout in orthopaedic surgeons: A review. ANZ J. Surg..

[46] Lawlor S.K., Low C.M., Carlson M.L., Rajasekaran K., Choby G. (2022). Burnout and well-being in otolaryngology trainees: A systematic review. World J. Otorhinolaryngol. Head Neck Surg..

[47] Govardhan L.M., Pinelli V., Schnatz P. (2012). Burnout, depression and job satisfaction in obstetrics and gynecology residents. Conn. Med..

[48] Cambridge Dictionary. https://dictionary.cambridge.org/us/dictionary/english/work-life-balance.

[49] Kim T.G. (2021). A Study on Work Intensity, Work-Life Balance, and Burnout among Korean Neurosurgeons after the Enactment of the Special Act on Korean Medical Residents. J. Korean Neurosurg. Soc..

[50] Nwachukwu I., Nkire N., Shalaby R., Hrabok M., Vuong W., Gusnowski A., Surood S., Urichuk L., Greenshaw A.J., Agyapong V.I.O. (2020). COVID-19 Pandemic: Age-Related Differences in Measures of Stress, Anxiety and Depression in Canada. Int. J. Environ. Res. Public Health.

[51] González-Sanguino C., Ausín B., Castellanos M.Á., Saiz J., López-Gómez A., Ugidos C., Muñoz M. (2020). Mental health consequences during the initial stage of the 2020 Coronavirus pandemic (COVID-19) in Spain. Brain Behav. Immun..

[52] Su T.-P., Lien T.-C., Yang C.-Y., Su Y.L., Wang J.-H., Tsai S.-L., Yin J.-C. (2007). Prevalence of psychiatric morbidity and psychological adaptation of the nurses in a structured SARS caring unit during outbreak: A prospective and periodic assessment study in Taiwan. J. Psychiatr. Res..

[53] Ozamiz-Etxebarria N., Dosil-Santamaria M., Picaza-Gorrochategui M., Idoiaga-Mondragon N. (2020). Stress, anxiety, and depression levels in the initial stage of the COVID-19 outbreak in a population sample in the northern Spain. Cad. Saude Publica.

[54] Maslach C., Leiter M.P. (2016). Understanding the burnout experience: Recent research and its implications for psychiatry. World Psychiatry.

[55] 2018 National Resident Survey (2018). Resident Doctors of Canada. https://residentdoctors.ca/wp-content/uploads/2018/10/National-Resident-Survey-2018-R8.pdf.

[56] Williams D., Tricomi G., Gupta J., Janise A. (2015). Efficacy of burnout interventions in the medical education pipeline. Acad. Psychiatry.

